# Temporal Stability of Signal Quality in Non-Contact Biopotential Electrodes

**DOI:** 10.3390/s25103077

**Published:** 2025-05-13

**Authors:** Antonio Stanešić, Luka Klaić, Dino Cindrić, Mario Cifrek

**Affiliations:** University of Zagreb Faculty of Electrical Engineering and Computing, 10000 Zagreb, Croatia; luka.klaic@fer.unizg.hr (L.K.); dino.cindric@fer.unizg.hr (D.C.); mario.cifrek@fer.unizg.hr (M.C.)

**Keywords:** capacitive electrodes, biopotentials, ECG, cECG, biomedical signals

## Abstract

Non-contact electrodes have garnered significant attention as an alternative non-invasive biopotential measurement method that offers advantages such as improved subject comfort and ease of integration into everyday environments. Despite these benefits, ensuring consistent signal quality over time remains a critical challenge, particularly in applications like electrocardiography (ECG), where accuracy and reliability are paramount. This study investigates the temporal stability of signal quality in non-contact biopotential electrodes, with a primary focus on ECG monitoring. Our measurements showed a significant change in the recorded signal quality during prolonged measurement periods, which impacts the integrity and reliability of the measurements. Furthermore, it significantly impacts any shorter (<10 min) consecutive measurements of influential parameters (such as properties of electrodes, dielectric, etc.) since it removes the crucial *ceteris paribus* principle: the signal may not change just due to the change in influential parameters, but also due to the passage of time. Through a series of controlled experiments, we analyze how factors such as temperature, pressure on the electrodes, and humidity influence signal quality over extended durations (10 min or more). The results demonstrate key insights into the temporal dynamics of non-contact electrode performance, identifying potential sources of signal degradation and avenues for mitigation.

## 1. Introduction

Measurement and analysis of biopotential signals has been a hot topic of research for decades, since medical procedures, especially those of diagnostic nature, rely heavily on biopotential monitoring. Electrocardiograms (ECGs) are the most frequently used biopotentials since they can be used for assessment of cardiovascular health and functions, as well as in exercise, stress, or fatigue monitoring [1,2,3,4,5].

Biopotentials can be measured via needle, gel, semi-dry, dry, and non-contact electrodes. Needle electrodes are extremely invasive and could potentially cause pain and discomfort, but they achieve high selectivity and get as close as possible to the signal source.

Surface electrodes (gel, semi-dry, dry, and non-contact) are significantly less invasive but require contact with the subject’s skin. Gel (also called wet) electrodes, such as Ag/AgCl electrodes, are the most commonly used since the conductive gel reduces the contact impedance of the skin–electrode interface, which results in a significantly higher quality of recorded signals with respect to the signal-to-noise ratio (SNR) compared with other types of surface electrodes. Dry electrodes are increasingly common while providing more flexibility and comfort, but also a signal of lesser quality in comparison to wet counterparts. Semi-dry electrodes are positioned as a means between the two previously described concepts, combining the advantages of both wet and dry electrodes [6,7], but still require immediate contact with the subject’s body. Recent studies have proposed non-contact electrodes as biomedical sensors that rely predominantly on capacitive coupling in order to acquire biopotentials from the subject’s body. Throughout the rest of the paper, “non-contact electrodes” term will be used to describe such electrodes, while “capacitive electrodes” or “non-contact capacitive electrodes” are also commonly used terms. In that sense, ECG recorded using such electrodes will be referred to as capacitive ECG (cECG). Such electrodes provide a minimally invasive interface that is more independent of skin condition and with little to no requirements for skin preparation. They can also be easily embedded into clothes, mattresses, etc., simplifying the measurement process, making it quicker, and enabling measurements in cases in which they would traditionally not be possible [8,9,10,11]. Furthermore, such electrodes do not require the application of the conductive gel. They are reusable and more comfortable, which reduces cumulative costs of use and waste generated. Subject safety is also improved since the electrodes are not in direct contact with the body, as opposed to conventional electrodes. Those advantages come with the burden of a lower SNR and new types of artifacts and interference sources.

Non-contact electrodes/sensors function primarily based on capacitive coupling between the electrode’s sensing surface and the human body’s surface, which forms a capacitor, with a dielectric in-between usually being fabric or clothes. The equivalent capacitance of such a capacitor is very small, in the range of a few picofarads [12], which can be increased by increasing the coupling area of the electrode. This, in turn, increases the susceptibility to artifacts, noise, and other interference sources. This tradeoff presents a typical non-contact electrode design challenge.

Non-contact electrodes are systems consisting of three key components:

1. Sensing surface, also known as detection disk, which represents the immediate interface toward the human body;
2. Preamplifier, which transforms the high impedance of the front end into low impedance for further amplification and processing;
3. Data acquisition system for digitization of the data, enabling further digital signal processing.

In recent works, authors primarily used sensing surfaces of rectangular [13,14] or circular [15,16,17,18,19] shapes of linear dimensions under 50 mm, such as in [16], where the diameter of the electrode was 25 mm, or in [12], where the surface was a square with sides of 45 mm in length. While smaller dimensions of the electrodes would produce results of lower quality (due to lower equivalent capacitance), they are potentially much more practical. Authors in [15] state that due to relatively weak capacitive coupling, the dimensions of the sensing area should be almost double in size compared with standard gel electrodes.

There are various preamplifier topologies, with the most basic being an operational amplifier in a simple voltage follower configuration. Such a solution is often used [18,20,21,22]. A minor addition to such a configuration is also commonly used [8,10,13,23], where a high-value resistor is used to ensure a pathway for the input capacitor to discharge [8,10,13,23], preventing the input voltage from drifting outside the common-mode voltage range (CMVR). More advanced topologies include bootstrap topology and transimpedance amplifier as described in [15], or the topology presented in [12], which is optimized for mitigating artifacts due to the movement of other persons and/or conductive objects in the vicinity of the subject. Preamplifiers commonly employ the LMP77xx series of amplifiers, such as LMP7715 or LMP7721 [24]. Those amplifiers are specifically chosen due to their very high input impedance (range over 1 TΩ), very low input capacitance (under 2 pF), high common-mode rejection ratio (CMRR), very low level of input bias current (20 pA maximum at ambient temperature), etc.

Acquisition systems that are commonly used to sample and digitize the signals from the preamplifiers in non-contact electrodes can be divided into two subgroups: general-purpose acquisition cards such as NI DAQ used in [21], and custom-made acquisition systems based on instrumentation amplifiers and analog-to-digital converters (either standalone or integrated into the microcontroller).

One of the key challenges in designing non-contact electrodes is noise and interference mitigation. Besides usual noise and interference sources (due to components, biopotential cross-coupling, and common mains voltage coupling), non-contact electrodes often behave like antennas that can sense people’s movements and conductive objects near the subject. There are multiple ways of combating such interference, such as shielding, capacitive-driven right leg (CDRL), and various software-based filters. Shielding and guarding (such as in [15]) can help with the directionality of the measurements, so that external electromagnetic interference is minimized. The guard ring is commonly placed around and below the sensing surface, thus encapsulating it in all directions except the one oriented toward the subject’s body. CDRL can mitigate common mains voltage coupling by orders of magnitude [25], while software filters, commonly based on adaptive filtering principles, can remove many interference sources at once, but often modify the useful signal substantially and are implemented a posteriori [26,27].

One potential problem, that authors [9,28] have noted, has been the question of the repeatability of the results, mainly due to varying surroundings, which can influence the measurements greatly. A systematic and comprehensive approach to non-contact electrodes seems to be a logical step toward solving such problems. Our research into this topic [29,30,31,32], up to this point, produced many measurements using various topologies of non-contact cECG electrodes. We have thus noticed a significant change in the recorded signal quality during prolonged measurement periods. There have been cases where the quality of the signal increases monotonously over time or has alternating phases of increase and decrease, even when the subject is completely still, and no other visible parameters are changing. This phenomenon greatly reduces confidence in the results of sequential measurements, since there is a new, hidden, invisible parameter (or multiple parameters) that influences the measurements, thus removing the necessary *ceteris paribus* principle required for reliable research. In practice, if one were to investigate the influence of certain parameters (such as fabric thickness, fabric dielectric permittivity, and detection surface area), measuring each parameter separately while maintaining controlled conditions would be impossible due to the presence of these unexplained temporal variations in signal quality. The results would be confounded by these hidden variables, making it difficult to isolate the true effect of each parameter under investigation.

In this work, a systematic analysis of the most probable hidden parameters has been carried out in order to investigate their influence on surface non-contact cECG measurements on real human subjects. These parameters are:

1. Sweat/micro-sweat;
2. Temperature or temperature gradients;
3. Change in the pressure on the sensor (also called “pressure” for the sake of simplicity and brevity);
4. Inherent issues of the non-contact electrode topology.

Perspiration (sweat), even in microscopic amounts (also known as micro-sweat), could alter the measurement by changing the dielectric properties, which has so far seldom been reported [26,33]. The impact of ambient RH on textile, in general, and cotton, in particular, has been discussed in [34], where it was found that DC electrical conductivity increases exponentially as ambient RH increases. In the context of non-contact electrodes, the beneficial impact of ambient RH on signal quality was noted in [35]. The beneficial impact of sweat on non-contact electrode cECG measurements has been demonstrated in [35,36,37,38]. Perspiration could change the coupling properties of the skin–electrode interface, making them predominantly ohmic, especially in the case of hygroscopic dielectrics like various types of cloths, which are also incidentally the most common dielectrics in wearable applications of non-contact electrodes. Temperature changes could introduce component value mismatch (especially in resistors) in various stages of the measurement chain, which could then directly impact the CMRR, leading to varying SNR. A change in the pressure on the sensor could alter the coupling area and impedance characteristics at the electrode–skin interface. It could compress the dielectric material, thus changing its effective thickness, or changing the relative area of the equivalent capacitor plates, which would alter the capacitance of the equivalent capacitor. If none of these parameters show any correlation with the SNR change during the measurements, the issue might lie in the non-contact electrode topology itself, due to the charging/discharging of the equivalent input capacitor of the skin–electrode interface or preamplifier parameters, such as input offset voltage and input bias current drift with respect to time and temperature, etc. All of these parameters should be measured without influencing the non-contact cECG measurements.

The rest of the paper is organized as follows: Section 2 surveys the developed measurement system and its components and describes the methodology and measurement protocol. Next, Section 3 brings the obtained results, while Section 4 presents a discussion of the results and positions them in the broader context of the existing research. Lastly, Section 5 delivers a conclusion.

## 2. Materials and Methods

The hidden parameters have been investigated by simultaneous synchronized measurement of the parameters with both non-contact cECG signal and ECG signal recorded using conventional gel electrodes. This enables precise calculation of SNR change in the non-contact cECG recordings by using the conventional gel electrode measurements as a reference standard and correlating them with the hidden parameters of sweat (humidity), temperature, and pressure on the sensor. The aim is to provide guidelines for more reliable and credible non-contact measurements, as well as give greater insight into non-contact cECG measurements, and biopotential measurements in general.

**Figure 1:**
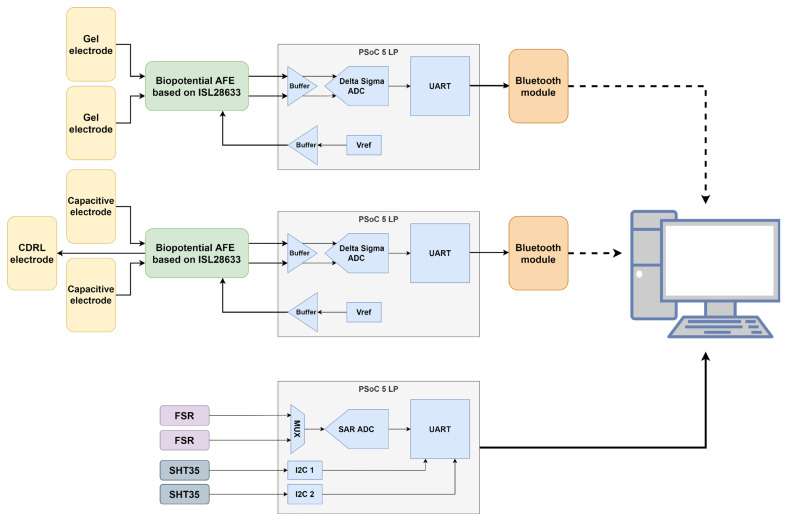
Complete measurement system overview.

The complete measurement system is shown in Figure 1.

Identical biopotential analog frontends (AFE) are used for interfacing non-contact electrodes and conventional gel electrodes. This is performed in order to have comparable signal characteristics and eliminate any variations that might arise from different electronic components or circuit designs. By maintaining consistent filtering, amplification, and digitization processes for both recording methods, any observed differences in signal quality can be more confidently attributed to the electrode–skin interface properties rather than to the measurement electronics.

The AFE consists of three stages:

1. An input differential low-pass filter—which serves as both a radiofrequency interference (RFI) filter and an anti-aliasing filter (AAF) with a corner frequency of 500 Hz;
2. An ISL28633 (Renesas Electronics Corporation, Tokyo, Japan) fully differential instrumentation amplifier [39];
3. A PSoC™ 5LP microcontroller (Infineon Technologies AG, Neubiberg, Germany) [40] that uses an internal Delta-Sigma analog-to-digital converter (ADC) for signal digitization.

This AFE has significantly improved CMRR in comparison to our previous AFE (used in [41]), while maximally preserving the phase of the ECG signal (due to having only one filtering stage). Preserving the phase of the ECG signal is crucial for accurately detecting waveform morphology, such as PQRST complexes, which are essential for clinical-grade diagnostics. The AFE is described in more detail in [42].

**Figure 2:**
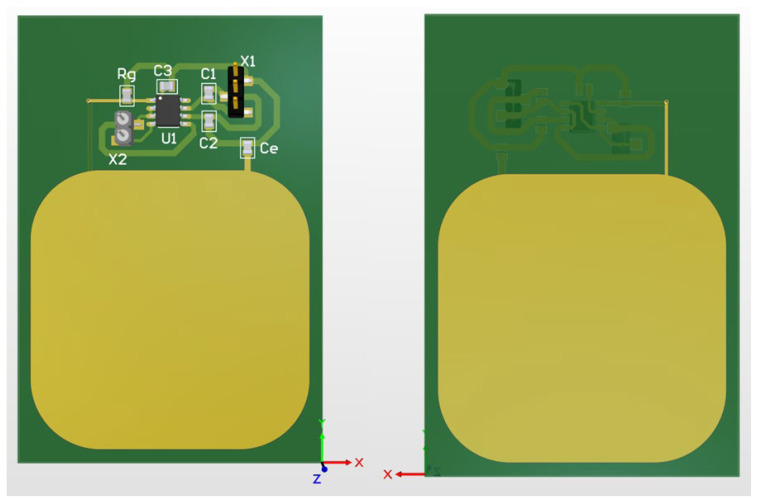
Three-dimensional model of used non-contact electrodes, front and back view. Green denotes portions covered with soldermask; while yellow denotes portions with exposed pads.

**Figure 3:**
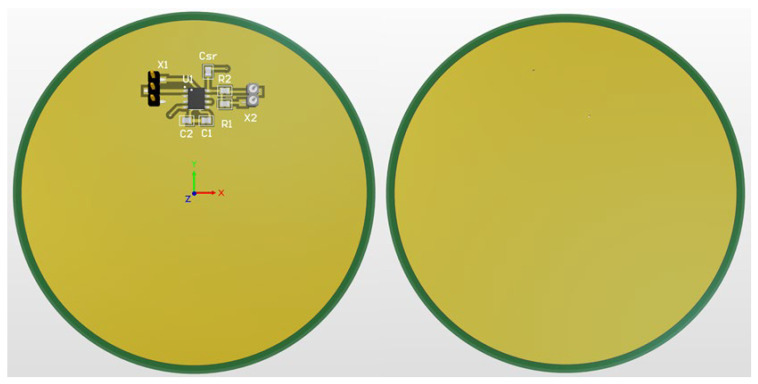
Three-dimensional model of capacitive right leg drive (CDRL) electrode, front and back view. Green denotes portions covered with soldermask; while yellow denotes portions with exposed pads.

The non-contact electrodes used in this research build upon work outlined in [12] and are shown in Figure 2, with several added modifications. Instead of the original rectangular sensor surface, we opted for rounded rectangles since this shape reduces electric field concentration at the corners, which happens due to the edge/corner effect, potentially improving signal quality and reducing noise [43]. We also incorporated shielded cables to better isolate the electrode connections from environmental noise and removed the solder mask from the sensing area to avoid the introduction of another dielectric. Each of the electrodes has a sensing area of approx. 20 cm^2^. We also implemented a CDRL electrode to enhance the CMRR, consistent with approaches described in [12,25]. The CDRL electrode (shown in Figure 3) is circular and has a coupling area of 80 mm in diameter. Initial measurements that led us to investigate this hidden parameter phenomenon have been performed using a set of non-contact electrodes without using a CDRL electrode, inspired by the work described in [15]. Since this phenomenon manifested across two electrode sets with different topologies, we can conclude that the observed effects are not artifacts specific to a particular electrode design but seem inherent to the non-contact measurement principle itself. Gel electrodes used in the research were disposable Cleartrace2 LT electrodes by ConMed, Largo, FL, USA (15 mm in diameter).

The platform that samples the three selected hidden parameters of skin humidity, temperature, and pressure on the sensor has been built upon the same microcontroller (PSoC™ 5 LP) as the AFE to simplify code generation and interfacing with the PC. The platform implements interfaces toward two force-sensing resistors (FSR) and two SHT35 precision temperature and humidity sensors [44].

**Figure 4:**
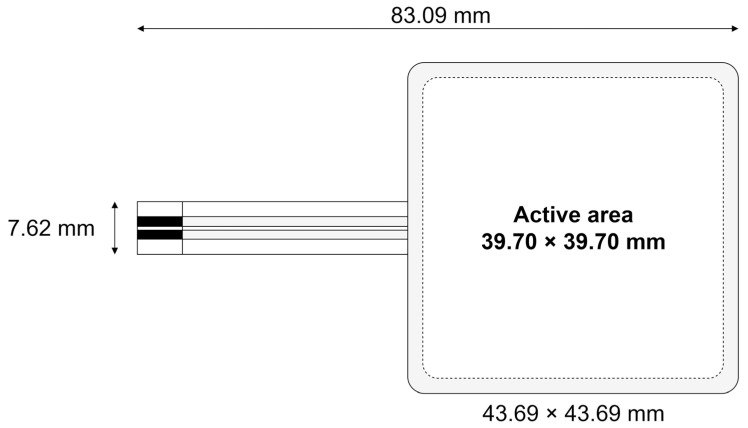
Chosen FSR and its dimensions (adapted from [45]). Dashed lines indicate the active sensing area (39.70 × 39.70 mm), while solid lines define the physical sensor dimensions (43.69 × 43.69 mm). The black region represents the solderable area.

Two identical FSRs are placed under the non-contact electrodes in order to measure pressure on the sensor during the measurements. We have chosen Ohmite (Warrenville, IL, USA) FSR01CE (shown in Figure 4) due to their near-identical dimensions to our sensing area (39.7 mm × 39.7 mm) and good characteristics (resistance from 1 Ω to 10 MΩ for 20 g to 5 kg loads, ±4% repeatability between part-to-part [45]). To interface the FSR, internal current digital-to-analog converters (DAC) have been used inside of the PSoC™ 5 LP to generate excitation current, while the voltage across the sensor has been sampled using the internal successive approximation register (SAR) ADC via an internal analog multiplexer. Thus, the whole interface is implemented within a single integrated circuit, eliminating the need for external components and reducing potential points of failure and mechanical setup while simplifying the overall system design.

**Figure 5:**
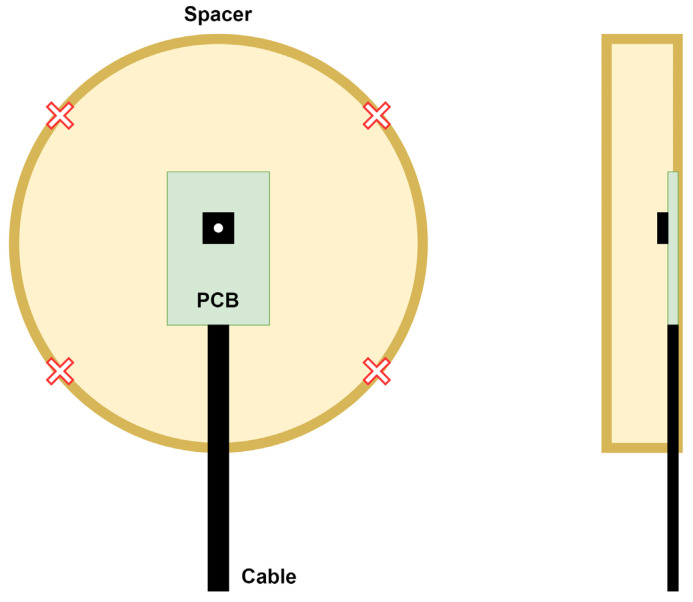
PVC spacer with SHT35 sensor embedded viewed (**left**) from the top and (**right**) from the side—each red X denotes the position of a 1 mm ventilation hole.

A simple printed circuit board (PCB) used for interfacing SHT35 has been developed—the board connects to the PSoC™ 5 LP microcontroller (which provides power supply) via I2C communication protocol. SHT35 (Sensirion AG, Stäfa, Switzerland) has been selected since it is fully calibrated, linearized, and temperature compensated, with typical accuracy of ±1.5% relative humidity (RH) and ±0.1 °C, and is offered in a very small dual flat no-leads (DFN) package of just 2.5 mm × 2.5 mm. This enabled us to have the whole sensor mounted on a PCB of just 10.0 mm × 15.0 mm. The sensor has been internally set for the high-reliability measurement option. While the temperature could be measured just by placing the sensor on the skin of the subject, skin humidity cannot be reliably and precisely measured using such an approach. Thus, we have devised a polyvinyl chloride (PVC) spacer (20 mm radius, 6 mm total inner height, shown in Figure 5), which would create a small semi-enclosed air pocket between the subject’s skin and the sensor, with specific “microclimate”, which can then be sampled for both temperature and relative humidity changes. The PVC spacer needs to be almost completely enclosed but also has some ventilation holes, which will disable the complete saturation of the air pocket with moisture. We empirically concluded that including four 1 mm diameter holes evenly spaced around the shell of the spacer provided enough sensitivity while preventing complete sensor saturation. While this approach does not give a precise sweat volume measurement, it can effectively detect significant changes in perspiration activity. Similarly, although the sensor does not measure skin temperature directly due to the physical separation introduced by the PVC spacer, it effectively captures temperature trends that correspond to skin-level changes. This allows for relative thermal monitoring while maintaining a non-intrusive measurement principle. Temperature readings can provide further context for the humidity, as increased perspiration generally correlates with rising skin temperature. A 3–5 mm height ensures the sensor remains outside the immediate thermal boundary layer of the skin (~1–2 mm).

To ensure proper operation of both the non-contact and gel electrode measurement, galvanic isolation has been implemented. The simplest way of attaining such a goal was to power each of the measurement systems from separate, galvanically isolated power supplies. The FSR and SHT35 sensor platforms are connected directly to the measurement laptop since neither FSR nor SHT35 are in direct contact with the subject’s skin. The gel electrodes do not influence the non-contact electrodes nor vice versa, and neither is affected by the sensor platform.

**Figure 6:**
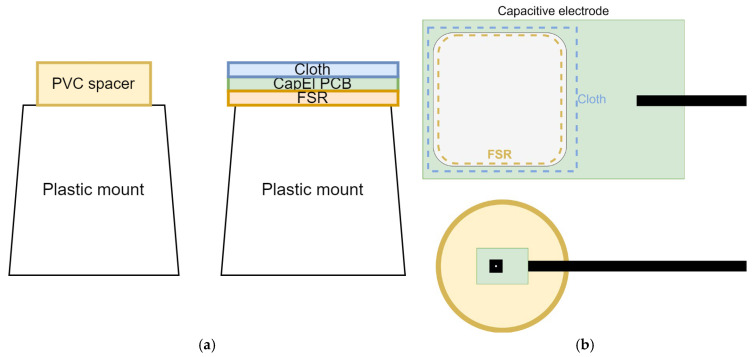
Mechanical setup viewed (**a**) from the side and (**b**) from the top.

**Figure 7:**
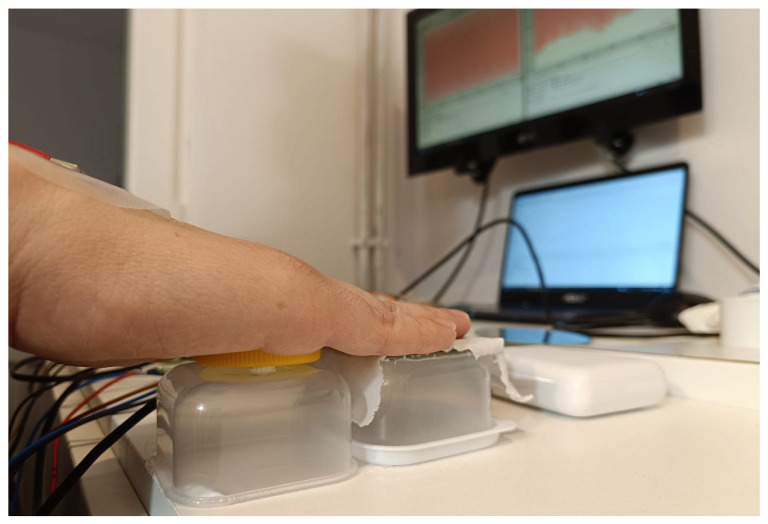
Placement of hands over the sensors and non-contact electrodes mounted on the plastic mounts (shown in Figure 6).

The mechanical layout is presented in Figure 6. Two plastic mounts are used for each hand—one mount has SHT35 inside of a PVC spacer, while FSR and the non-contact electrode are placed on another one. The mounts are dimensioned so that the top surface of the PVC spacer aligns with the coupling area of the non-contact electrode, ensuring that the user’s palm rests evenly on both elements (Figure 7). This co-planar design minimizes posture-related bias and improves the repeatability of contact. These are fixed in place so that the whole measurement setup is not moving (neither relatively nor absolutely) during the measurement. This also helps preserve the *ceteris paribus* principle necessary for this research. The strip of cotton cloth (about 0.3 mm thick) is placed over the non-contact electrode to serve as a dielectric.

Each measurement is performed according to the following protocol:

1. The subject is informed about the measurement procedure, reasons for collecting each of the recorded data and the whole context of the measurement, and gives consent if agrees with the presented conditions;
2. The subject is seated in the seat with CDRL placed under the subject’s right leg;
3. The skin on the subject’s wrists, where the gel electrodes will be placed, is cleaned and prepared for the adhesion of gel electrodes according to the common practice for such measurements;
4. Gel electrodes are placed on each of the wrists of the subject;
5. The subject places hands over the sensors according to Figure 7;
6. The measurement assistant starts the measurement for each of the sensors and moves at least 2 m away from the subject to avoid influencing the measurements. The subject is instructed to remain as still as possible while breathing normally;
7. After the effect has been clearly manifested (at least 10 min) and the subject starts to feel discomfort due to lack of movement, the measurement is completed, and electrodes are disposed of while the data are being saved. The pair of cloths was replaced for a new measurement.

Measurements were received on the laptop via Bluetooth Serial Port Profile (SPP) and the communication (COM) ports. The data have been saved along with standard Unix/Posix timestamps in order to allow easier synchronization between the measurement systems. The processing of the data was performed in the Mathworks MATLAB R2024a environment. Recorded ECG (and cECG) data were high-pass filtered using 0.5 Hz as a corner frequency in order to remove motion artifacts, which are not a primary concern of this research. ECG data are recorded with a sampling frequency of 1000 Hz for both gel and non-contact electrodes, while both channels of temperature, humidity, and pressure on the sensor were sampled at 2 Hz since these data are not fast-changing.

In total, measurements have been performed on 7 healthy, willing subjects. The demographic characteristics of the subjects, including age, sex, and BMI, are summarized in Table 1. A total of over 4 h of measurements have been recorded. One of the subjects (F/58) has a condition of semi-voluntary palmar hyperhidrosis, while other participants have no declared history of excessive hand sweating or related dermatological conditions.

## 3. Results

Typical results from our extended measurement sessions are presented in this section to illustrate the data processing methodology and analytical approach. Visual representations of results from one representative dataset will be shown to highlight signal characteristics, processing steps, and derivation of key parameters. This comprehensive examination of a single measurement provides context for interpreting the complete set of results.

### 3.1. Stability of Gel Electrodes vs. Non-Contact Electrodes

**Figure 8:**
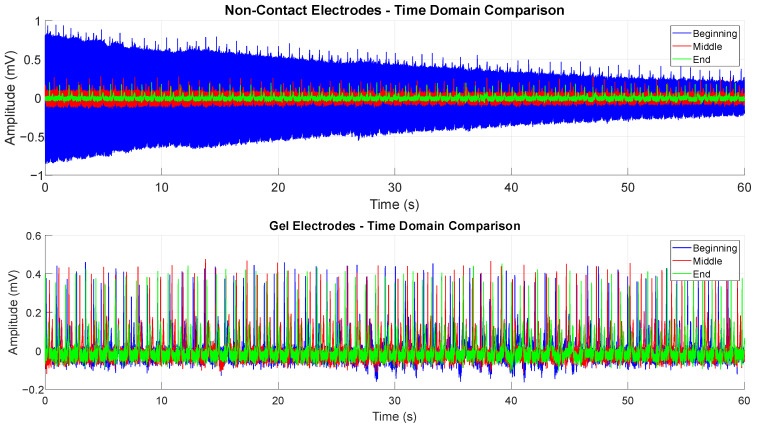
Demonstration of the temporal stability of gel electrodes and temporal instability of non-contact electrodes in the time domain.

Prior to investigating the impact of various parameters on SNR and temporal stability of non-contact electrodes generally, it is important to establish that the phenomenon occurs only when using non-contact electrodes, and not when conventional gel electrodes are used. This is quite evident from the time domain, as shown in Figure 8. One-minute segments are shown, from the beginning of the measurement (30 s to 90 s), the middle of the measurement (150 s to 210 s), and near its end (370 s to 430 s). The whole measurement lasts approximately 10 min. It is clear that in the first minute, the signal changes quite rapidly, with overall noise decreasing substantially toward the end of the segment. The middle segment shows a much more recognizable ECG signal, which still shows a small, but noticeable increment in SNR at the end of the interval in comparison to the start of the segment. The ending segment is the one most similar to conventional ECG and shows no further progress during the whole segment duration. In comparison to non-contact electrodes, segments pertaining to gel electrodes do not show significant changes in SNR during either of the three segments. The time segments are identical for both non-contact and gel electrodes, with their time bases aligned.

**Figure 9:**
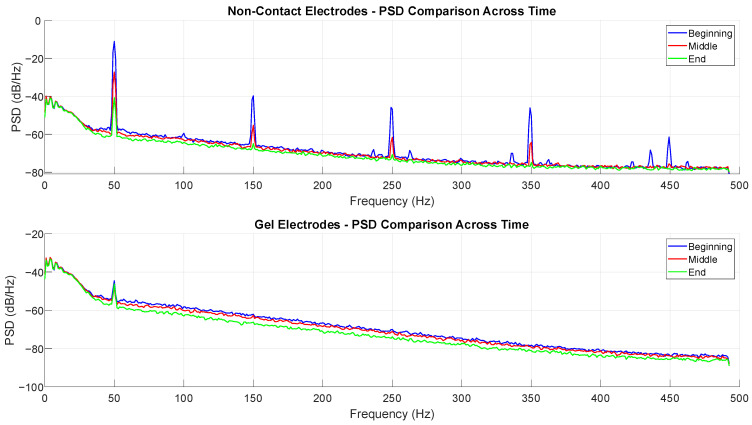
Demonstration of the temporal stability of gel electrodes and temporal instability of non-contact electrodes in the frequency domain.

Similarly to the time domain, the temporal instability of non-contact electrodes and the temporal stability of gel electrodes can also be seen in the frequency domain. The top graph in Figure 9 shows a significant change in frequency content during the measurement, primarily through the gradual diminishment of 50 Hz mains interference and its harmonics. The primary ECG spectrum, located in the frequency band of approx. 0.5–40 Hz, is not significantly changing during the measurement. On the other hand, the bottom graph shows little to no difference in frequency content during the three segments. The segments in question are the same as in Figure 8.

### 3.2. SNR Computation

In order to compare the change in signal quality to other measured parameters, it is necessary to compute SNR through time and compare it to the parameters. The SNR is computed by dividing the signal power by the noise power and is typically expressed in decibels (dB). The ECG signal recorded using the gel electrodes is used as the “gold standard” in computing the SNR, except in cases in which the gel electrodes detached during the measurement due to perspiration or reduced adhesion. In those measurements, SNR was computed by isolating the noise and signal frequency components in the spectrum (ECG is mainly concentrated around 0.5 Hz to 40 Hz band, while the noise is mainly 50 Hz and its harmonics. This distinction enabled utilization even of those measurements where electrode detachment occurred.

**Figure 10:**
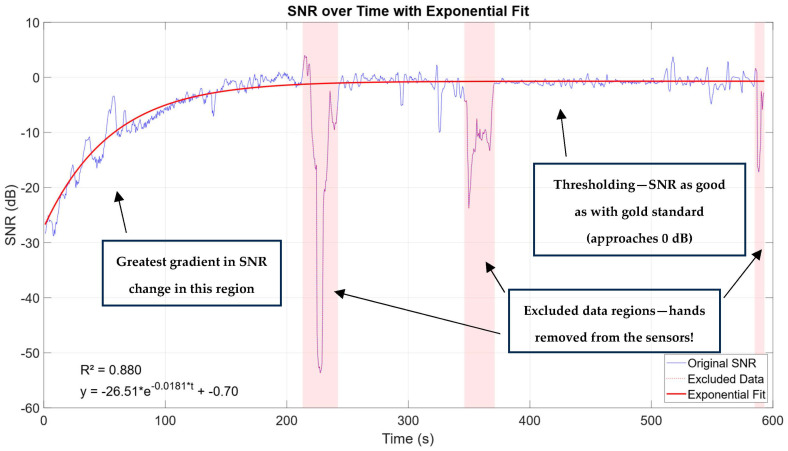
Graph showing a change in SNR over time, with excluded data segments colored red and exponential fit. In the lower-left corner, the goodness of fit and the fitted curve parameters are displayed. Annotations are added to highlight the region of the greatest gradient in SNR change, thresholding when SNR approaches gold standard levels and excluded data regions.

**Figure 11:**
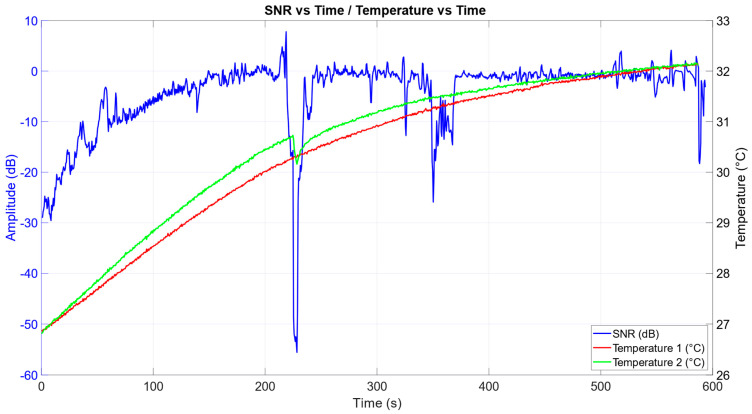
Graph showing a change in SNR over time compared with measurements from the two temperature sensors. Excluded regions have been deliberately left in this graph to illustrate the removal of one hand from the sensors for a brief moment (about 4 s).

**Figure 12:**
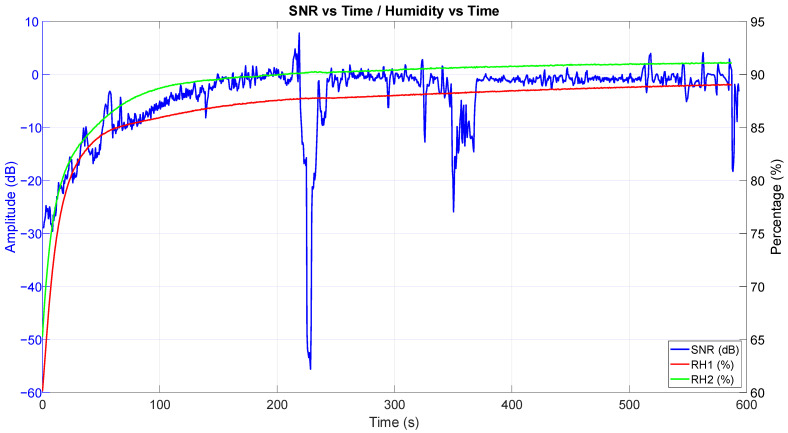
Graph showing a change in SNR over time compared with measurements from the two relative humidity sensors.

**Figure 13:**
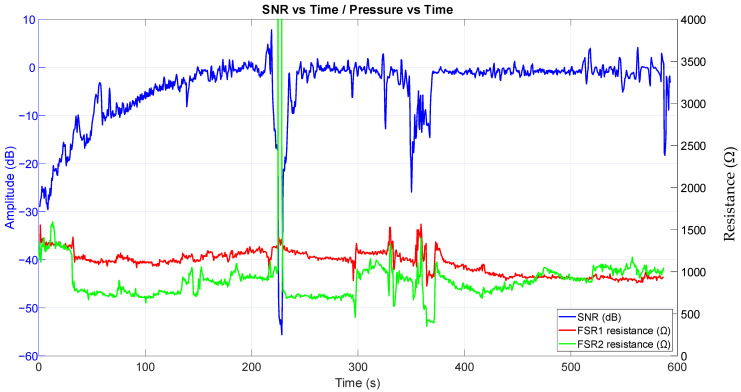
Graph showing a change in SNR over time compared with measurements from the two FSR sensors for detecting pressure on the sensor. Excluded regions have been deliberately left in this graph to illustrate the removal of one hand from the sensors for a brief moment.

SNR was computed over the whole length of measurement, in windows of 1 s and 6 s with 50% overlap in both cases. Narrower windows offer a better temporal resolution, while wider windows can provide greater frequency resolution, particularly for lower-frequency components of the ECG signal. A window size of 1 s was used for generating Figure 10, Figure 11, Figure 12 and Figure 13, while a window size of 6 s was used for the rest of the calculations.

Some measurements had small segments of larger artifacts, like the one shown in Figure 10. On three separate occasions (with times around 213–242 s, 346–371 s, and 585 s until the end of the measurement) the subject significantly moved his hands or removed one of them from both the sensor and the electrode completely (which is also noticeable in subsequent SNR vs. parameter graphs). These data were excluded from the exponential fit calculation, as well as from the subsequent correlation calculations and scatter plots. The exponential fit was used since it was observed that the SNR vs. time graph follows an exponential curve relationship. This can be attributed to two factors: first, the coupling behavior gradually becomes predominantly ohmic over time; second, the SNR is calculated relative to the “golden standard”, causing the SNR to asymptotically approach a stable value as coupling transitions from predominantly capacitive to predominantly ohmic. In other words, the impedance is increasingly dominated by its ohmic component, whose frequency dependence is negligible at the frequencies of interest in this study.

### 3.3. SNR vs. Temperature

In Figure 11, SNR change over time is shown together with the data from the two temperature sensors. There is a slight temperature difference in the middle of the measurement (about 0.4 °C at most). Temperatures in the air pockets are monotonously rising, albeit slower as the measurement progresses. The green line, depicting data from the second sensor, shows the precise moment at which the subject removed his hand from both the sensor and the non-contact electrode. The gel electrode stayed attached to the subject.

### 3.4. SNR vs. Relative Humidity

In Figure 12, along with SNR change over time, the data from the two relative humidity sensors are shown. Humidity in the air pockets is also monotonously rising, but much slower as the measurement progressed, and the curves look very similar to the SNR vs. time curve (Figure 10).

### 3.5. SNR vs. Pressure on the Sensor

Figure 13 depicts the change in time of the SNR, along with the pressure on the sensor estimated through the two FSR sensors. Greater pressure on the sensor lowers the resistance of the FSR. While there is variation in pressure on the sensor, especially in the second FSR, greater artifacts are introduced only when there is a substantial change in the pressure.

### 3.6. Scatter Plots and Correlations

Since some of the aforementioned graphs show similarity with the SNR vs. time graph (Figure 10 and Figure 12), a correlation calculation would quantify both the degree of correlation between the parameters and the SNR, as well as the statistical significance of the calculated correlation. Scatter plots can help with identifying potential non-linear relationships and detect any outliers or clusters that might influence the correlation metrics. The color-coded temporal dimension adds an additional layer of information, revealing how these relationships evolved throughout the measurement period.

**Figure 14:**
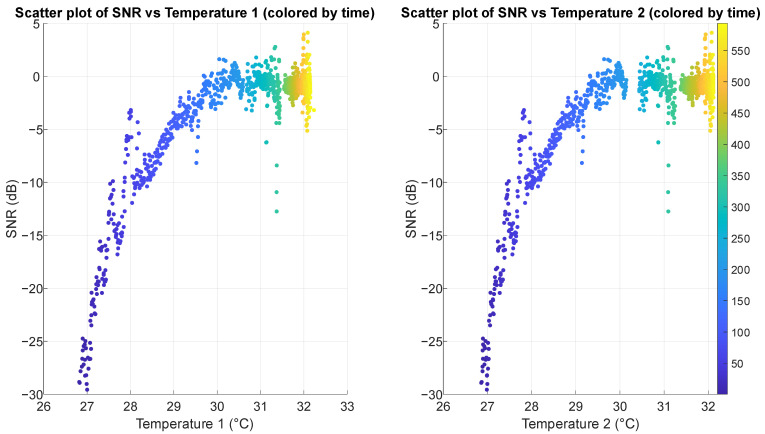
Scatter plots illustrating the relationship between SNR and temperature in the air pocket gathered from two sensors, with points colored by measurement time (in seconds). Both plots show a strong positive correlation.

**Figure 15:**
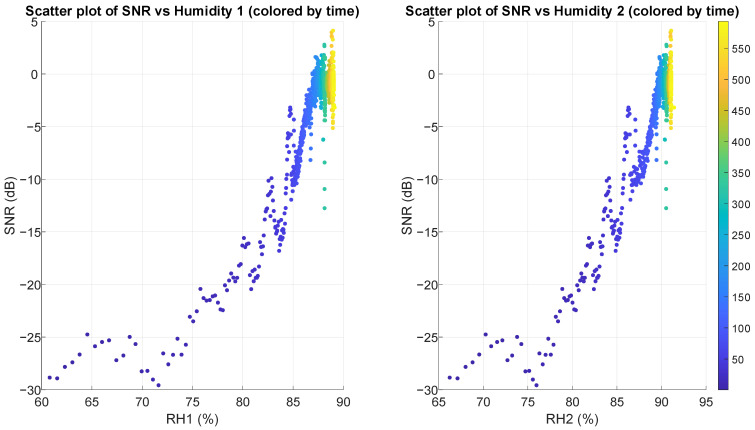
Scatter plots illustrating the relationship between SNR and relative humidity in the air pocket gathered from two sensors, with points colored by measurement time (in seconds). Both plots show a very strong positive correlation.

**Figure 16:**
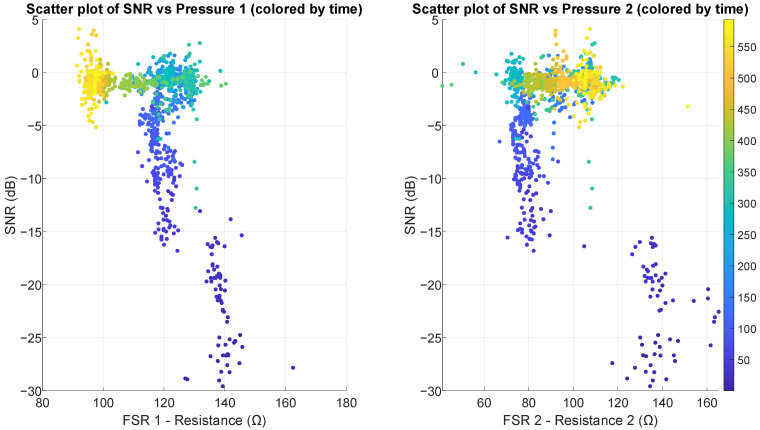
Scatter plots illustrating the relationship between SNR and pressure on the sensor using two FSR sensors, with points colored by measurement time (in seconds). The left plot shows a moderate negative correlation, while the right plot shows no clear correlation or pattern.

Scatter plots in Figure 14 and Figure 15 reveal strong positive correlations between SNR and both temperature and humidity, with clear non-linear patterns. As the temperature in the air pocket increases from 26 °C to 33 °C, SNR improves dramatically from around −30 dB up to +5 dB. Similarly, as relative humidity increases from 60% to 90%, SNR shows consistent improvement. Regarding pressure on the sensor, scatter plots (shown in Figure 16) demonstrate a weak to moderate negative correlation between FSR measurements and SNR, particularly visible in FSR1. For FSR1, as resistance increases from 80 Ω to approximately 160 Ω, SNR decreases substantially. The relationship appears more scattered for FSR2, explaining its correlation coefficient (in Table 2).

Relative humidity (RH1 and RH2) in the air pocket demonstrates the strongest positive correlation with SNR, with Pearson coefficients exceeding 0.93 and Spearman coefficients exceeding 0.75 (strong correlation). Temperature measurements show similarly strong positive correlations. Calculated coefficients for these parameters (temperature and humidity) are also highly statistically significant (*p*-value under 10^−100^).

Analogously, the correlation coefficients for all subjects have been computed (Table 3).

### 3.7. Parameter Isolation Tests

Since the rise in the temperature and the rise in the relative humidity in the air pockets are physically correlated, it was necessary to perform additional measurements to distinguish which parameter is the one that is primarily influencing the change in SNR over time. This was performed in three ways:

- Test of non-hygroscopic dielectric;
- Changing the initially used cloth for a new, dry cloth after the measurement has entered the steady state (SNR at 0 dB or better, after approx. 10 min);
- Testing SNR of a slightly damp cloth.

**Figure 17:**
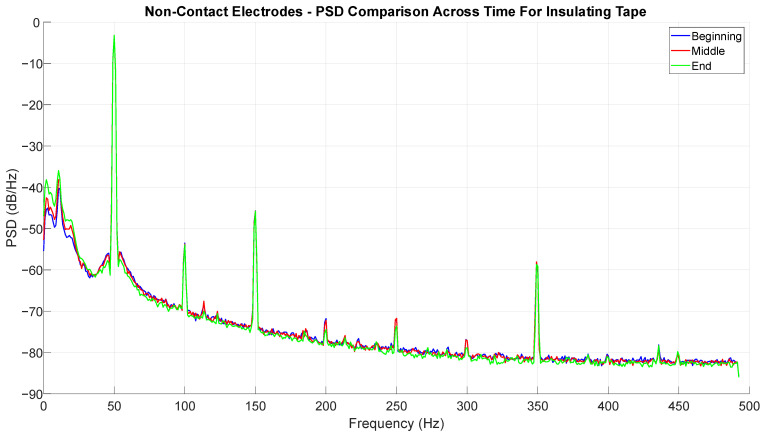
Demonstration of temporal stability of non-contact electrodes when using a non-hygroscopic dielectric.

While recording the cECG using non-contact electrodes with a thin layer of non-hygroscopic dielectric (such as insulating tape), the phenomenon does not manifest, as shown in Figure 17. The SNR is very low and remains such for the duration of the measurement. The ECG signal is visible in the 0.5 Hz–40 Hz range, but the 50 Hz mains hum and its harmonics are very significant. There is no significant change in the spectrum during the three 60 s segments taken from the beginning, middle, and end of the measurement.

This is confirmed by exchanging of the cloth under test after approximately 10 min for a new, dry cloth—the SNR changes negatively by about 20–40 dB, almost equaling the starting conditions.

Finally, when testing the SNR of a slightly damp cloth, sprayed using a sprayer bottle from a distance of 1 m, the immediate SNR change of about +20 dB is achieved.

## 4. Discussion

The scatter plots, correlation coefficients, and comparison of SNR changes over time with the measured parameters in the time domain indicate that humidity is the most correlated parameter with the SNR. The relationship is positive, and as humidity rises, so does the SNR. The correlation is highly statistically significant. The fact that the Spearman coefficient is typically notably lower (except in subjects B and E, where both coefficients are similar) suggests that there may be some non-monotonic aspects to the relationship. This likely occurs due to a threshold effect—after a particular value of relative humidity (in our measurements around 85–90%), a transition to predominantly resistive (ohmic) coupling occurs, which increases and stabilizes the SNR.

Temperature and humidity are commonly physically correlated and greater temperature can also cause perspiration. Even though temperature also shows a significant correlation with the SNR, parameter isolation tests helped with identifying the root cause. Even though it is not possible to sense the dampness of the cloth, some moisture still persists and significantly influences the SNR. It is likely that microperspiration occurs, which moves coupling from predominantly capacitive to predominantly ohmic. This, in turn, significantly increases SNR. This supports the conclusions stated in [26], as well as the necessity of the “stabilization period” of up to 15 min mentioned in [33].

Another perspective can be considered by modeling the skin–electrode interface as a simplified equivalent electrical circuit: a parallel connection of a resistor (R) and capacitor (C) [46]. This R–C model accounts for both resistive and capacitive impedance components at the interface. An increase in humidity would primarily reduce the resistive component, thus leading to an overall increase in the SNR.

To explain the influence of pressure on the sensor, it is necessary to visualize the impact of its increase. If the hand is barely touching the sensor, the pressure has low values (FSR resistance values are high) and the coupling is reduced. As pressure on the sensor increases, the more surface of the skin covers the sensor area and the flatter the surface of the skin becomes. After the skin covers the whole sensor area and is as flat as possible, no further increase in SNR can be achieved by increasing the pressure on the sensor. That would explain the thresholding and generally weak and inconclusive correlation between SNR and the pressure on the sensor.

While simultaneous impedance and phase tracking could offer complementary insights, such measurements were excluded to preserve the non-contact nature of the setup and avoid introducing galvanic interference that could confound the analysis. Moreover, impedance variations are a symptom—not the root cause—of the capacitive-to-ohmic coupling transition; thus, they were not the focus of this investigation.

## 5. Conclusions

The goal of this research was to investigate hidden parameters that influence the temporal stability of SNR in non-contact electrodes. This goal was successfully achieved, and moisture/humidity is recognized as the key parameter influencing such SNR trends. Additional considerations regarding pressure exerted on the sensor, as well as the temperature, have been made based on the measurements and their subsequent analysis. In order to have consistent measurements of various parameters (as well as for the comparison of interfaces, frontends, and non-contact electrode topologies), humidity should be monitored or the measurements should be significantly longer (at least 15 min), so that thresholding occurs, and SNR stabilizes. The usage of non-hygroscopic and non-porous materials as dielectrics is also a good strategy, as these are not impacted by the moisture generated from microperspiration. These approaches should ensure reliable measurements with non-contact electrodes in the future. Future studies could explore the same phenomenon using flexible non-contact electrodes to develop a deeper understanding of the effects of pressure and distance between the body and the electrode in the context of substrate rigidity and adapting the electrode to body curves.

## Figures and Tables

**Table 1:** Demographic characteristics of the subjects, including age, sex (F—female, M—male), and body mass index (BMI).

	Age	Sex	BMI [kg/m^2^]
Subject A	29	M	23.9
Subject B	29	F	23.1
Subject C	55	M	28.7
Subject D	54	F	24.0
Subject E	58	F	22.5
Subject F	66	M	30.0
Subject H	21	F	20.7

**Table 2:** Calculated correlation coefficients (Pearson and Spearman) for each of the sensors. R-value represents the correlation coefficient, while *p*-value represents the statistical significance of the correlation. Values of *p* = 0 indicate extremely small *p*-values (*p* < 10^−100^). RH denotes relative humidity in the air pocket, Temp denotes temperature in the air pocket, while FSR denotes the resistance of the FSR sensor corresponding to the pressure exerted on the electrode.

	RH 1	RH 2	Temp 1	Temp 2	FSR1	FSR2
Pearson r	0.930	0.955	0.833	0.799	−0.500	−0.002
Pearson *p*	0	0	0	0	<0.0001	0.947
Spearman r	0.758	0.757	0.758	0.758	−0.400	0.117
Spearman *p*	0	0	0	0	<0.0001	0.0003

**Table 3:** Calculated correlation coefficients (Pearson and Spearman) for each of the sensors, for each of the subjects. The R-value represents the correlation coefficient, while *p*-value represents the statistical significance of the correlation. Values of *p* = 0 indicate extremely small *p*-values (*p* < 10^−100^). RH denotes relative humidity in the air pocket, Temp denotes temperature in the air pocket, while FSR denotes the resistance of the FSR sensor corresponding to the pressure exerted on the electrode.

		RH 1	RH 2	Temp 1	Temp 2	FSR1	FSR2
Subject A	Pearson r	0.930	0.955	0.833	0.799	−0.500	−0.002
	Pearson *p*	0	0	0	0	<0.0001	0.947
	Spearman r	0.758	0.757	0.758	0.758	−0.400	0.117
	Spearman *p*	0	0	0	0	<0.0001	0.0003
Subject B	Pearson r	0.785	0.804	0.969	0.957	−0.155	−0.668
	Pearson *p*	0	0	0	0	<0.0001	0
	Spearman r	0.820	0.820	0.820	0.820	−0.266	−0.636
	Spearman *p*	0	0	0	0	<0.0001	0
Subject C	Pearson r	0.946	0.976	0.911	0.892	−0.601	−0.234
	Pearson *p*	0	0	0	0	0	<0.0001
	Spearman r	0.755	0.703	0.754	0.756	−0.534	−0.333
	Spearman *p*	0	0	0	0	<0.0001	<0.0001
Subject D	Pearson r	0.958	0.896	0.916	0.920	0.138	−0.395
	Pearson *p*	0	0	0	0	<0.0001	<0.0001
	Spearman r	0.793	0.704	0.793	0.793	0.043	−0.414
	Spearman *p*	0	0	0	0	0.147	<0.0001
Subject E	Pearson r	0.815	0.829	0.856	0.866	0.085	−0.135
	Pearson *p*	0	0	0	0	0.005	<0.0001
	Spearman r	0.867	0.873	0.872	0.873	0.194	−0.117
	Spearman *p*	0	0	0	0	<0.0001	0.0001
Subject F	Pearson r	0.883	0.812	0.904	0.920	−0.392	−0.167
	Pearson *p*	0	0	0	0	<0.0001	<0.0001
	Spearman r	0.809	0.782	0.811	0.813	−0.749	−0.131
	Spearman *p*	0	0	0	0	0	<0.0001
Subject G	Pearson r	0.888	0.982	0.940	0.820	−0.237	−0.115
	Pearson *p*	0	0	0	0	<0.0001	0.0009
	Spearman r	0.688	0.640	0.688	0.641	−0.265	−0.171
	Spearman *p*	0	0	0	0	<0.0001	<0.0001

## Data Availability

The datasets presented in this article are not readily available due to legal and privacy concerns. Requests to access the datasets should be directed to the corresponding author.

## Abbreviations

The following abbreviations are used in this manuscript:

ECG	electrocardiography
SNR	signal-to-noise ratio
cECG	capacitive electrocardiogram
CMVR	common-mode voltage range
CDRL	capacitive driven right leg
CMRR	common-mode rejection ratio
AFE	analog frontend
RFI	radiofrequency interference
AAF	anti-aliasing filter
ADC	analog-to-digital converter
FSR	force sensing resistor
DAC	digital-to-analog converters
SAR	successive approximation register
PCB	printed circuit board
RH	relative humidity
DFN	dual flat no-leads (package)
PVC	polyvinyl chloride
SPP	(Bluetooth) Serial Port Profile
COM	communication port
